# 304 A Novel Burn Education Program in a New Comprehensive Burn Center

**DOI:** 10.1093/jbcr/irad045.279

**Published:** 2023-08-29

**Authors:** Keisha M ONeill, Kathleen Hollowed, Steven A Kahn, Aaron Lesher, Tiffany Smith

**Affiliations:** South Carolina Burn Center at MUSC, Summerville, South Carolina; South Carolina Burn Center at MUSC, Charleston, South Carolina; The South Carolina Burn Center/Medical University of South Carolina, Charleston, South Carolina; South Carolina Burn Center, Charleston, South Carolina; South Carolina Burn Center at MUSC, Charleston, South Carolina

## Abstract

**Introduction:**

In 2020 a large academic hospital with an existing pediatric burn program expanded its services to take adults. Prior to opening the comprehensive center, training and education for the nursing staff was designed and implemented, in amalgamation with the existing pediatric education program. This study describes and evaluates the implementation and efficacy of a novel burn nursing education program, established using the American Burn Association’s (ABA) nursing competencies and Kolb’s Experiential Learning Model.

**Methods:**

For the purpose of this study Cohort 1 (C1) is defined as 83 nurses hired prior to 2021 and Cohort 2 (C2) is defined as 304 nurses hired after 2022.

Didactic and simulated education sessions were created and presented in tandem for both initial and ongoing competency. Didactic sessions were evaluated in two cohorts because the program content was redesigned after 2021. C1 was evaluated using a pre-posttest, whereas, C2 was evaluated using only a post-test. Simulated content was evaluated using the verification in practice method with a check off sheet created using institutional wound care guidelines. Course efficacy was evaluated by attendees using a 1-5 Likert scale.

Test scores from 2020-2022 were compared using a T-test with a p-value of < 0.05 considered significant.

**Results:**

To date 387 nurses have completed a written exam, been validated in practice, and if in C1, completed an annual ongoing burn competency activity. In 2020, C1 showed a statistically significant increase in pre and posttest scores from 66.87%±9.36 to 88.26%±7.90, p=0.000015. When C1 was retested in 2021 using the same posttest, the scores were 80%±1.73 in 2020 to 84.33%±3.84 in 2022, p=0.36. The mean score for posttests for C2 in 2022 was 88%.

Course evaluations returned by 155 nurses (77%) in 2020 indicated the course was too long, and some of the information was exorbitant for nursing practice. Therefore, in 2021 the course was redesigned. An additional 251 (65%) nurses have taken the course since, and have reported a mean of 4.5/5 for course efficacy and 4.9/5 for an increase in nursing confidence to care for the burn patient population.

**Conclusions:**

The education program resulted in significantly higher posttest scores in C1 during the 2020-2021 period showcasing an increase in initial burn knowledge. Although the scores for C1 in 2022 were not statistically significant, the increase in mean scores shows the nurses not only retained the initial information, but built on it throughout the year with the addition of clinical practice. Nurses in C1 continue to practice at high levels and train nurses in C2 who reported the course was critical to their understanding of burn care. Nurses in both C1 and C2 will be required to complete an annual burn competency module to ensure ongoing competency in burn care.

**Applicability of Research to Practice:**

Nursing Education

